# *Heterorhabditis bacteriophora* Extracellular Vesicles Alter the Innate Immune Signaling in *Drosophila melanogaster*

**DOI:** 10.3390/genes16060613

**Published:** 2025-05-22

**Authors:** Duarte Toubarro, Eric Kenney, Christa Heryanto, Sreeradha Mallick, Nelson Simões, Ioannis Eleftherianos

**Affiliations:** 1 CBA and Faculty of Sciences and Technology, University of Azores, Rua Mãe de Deus no13, 9500-321 Ponta Delgada, Portugal; duarte.nt.tiago@uac.pt (D.T.); nelson.jo.simoes@uac.pt (N.S.); 2 Infection and Innate Immunity Laboratory, Department of Biological Sciences, The George Washington University, Washington, DC 20052, USA; etkenney2@gmail.com (E.K.); christa.heryanto@nih.gov (C.H.); sreeradha.mallick@gwmail.gwu.edu (S.M.)

**Keywords:** *Drosophila*, *Heterorhabditis*, infection, immune signaling, innate immunity, immune gene expression

## Abstract

**Background:** *Heterorhabditis bacteriophora* entomopathogenic nematodes are commonly used in agricultural practices for the biological control of insect pests. These parasites are also used in basic research for unveiling the molecular basis of nematode parasitism in relation to the insect anti-nematode response. We have recently shown that *H. bacteriophora* excreted–secreted products reduce the expression of the antimicrobial peptide gene *Diptericin* in *Drosophila melanogaster*, which increases fly mortality due to enhanced propagation of the mutualistic bacteria *Photorhabdus luminescens*. However, the effect of entomopathogenic nematode extracellular vesicles (EVs) on the insect host defense remains unknown. **Methods:** Here, we injected adult flies with *H. bacteriophora* EVs and used quantitative RT-PCR together with gene-specific primers to analyze the activity of immune-related signaling pathways. **Results:** We found that *H. bacteriophora* EVs are lethal to *Drosophila melanogaster,* and they downregulate the expression of *Attacin*, *Cecropin,* and *Prophenoloxidase 3* in adult flies. **Conclusions:** These findings build on previous knowledge and strengthen the notion that *H. bacteriophora* entomopathogenic nematodes release a variety of effector molecules to modify the insect’s innate immune signaling. This information is important because it contributes toward clarifying the molecular interplay between entomopathogenic nematode components and the host’s innate immune system.

## 1. Introduction

Entomopathogenic nematodes (EPNs) are parasitic nematodes that have evolved a mutualistic relationship with Gram-negative bacteria and are capable of locating and invading insects [1]. The remarkable efficiency of EPNs in causing insect disease renders them excellent biological control agents for the management of agricultural insect pests and disease vectors as well as outstanding research tools for understanding the molecular basis of nematode parasitism [2,3].

*H. bacteriophora* nematodes are associated with *P. luminescens* bacteria, and their relationship is highly specific [4]. The bacteria are localized in the gut of third-stage (L3) infective juveniles, which are found in the soil, and they are similar to the dauer juvenile stage of the free-living nematode *Caenorhabditis elegans*. The infective juvenile is in the non-feeding developmental stage, and it can survive for several months without a suitable insect host. *H. bacteriophora* infective juveniles develop into self-fertilizing hermaphroditic adults in the first generation, and succeeding generations consist of males, females, and hermaphroditic individuals. When space and food resources are consumed, a new generation of non-feeding infective juveniles exit the insect cadaver and disseminate into the soil to search for another suitable host to repeat their lifecycle [5,6].

The success of EPNs as potent insect pathogens is largely due to their ability to produce and release a cocktail of molecules with pathogenic properties and factors that undermine the insect’s innate immune system [7,8,9]. The immunomodulatory capacity of EPNs allows them not only to survive and migrate within the host but also to cause a powerful infection, which is characterized by extensive damage to insect tissues [10]. In particular, the excreted–secreted products of *H. bacteriophora* nematodes have the capacity to interfere with the signaling activity of the immune deficiency (Imd) pathway of the fruit fly *D. melanogaster*, as well as the phenoloxidase response of the waxworm *Galleria mellonella* [11,12].

Extracellular vesicles (EVs) have emerged as pivotal components of excretory–secretory (ES) products in helminths, facilitating intricate host–parasite interactions [13]. EVs have been recognized as key mediators of host–parasite communication and infection processes, but the presence of EVs in the excretory–secretory products of *H. bacteriophora* remains understudied [13,14]. Accumulating evidence indicates that EVs are key molecules in parasitic helminths, regulating the communication between parasites and the interaction with the host [14]. Given their immunomodulatory potential, characterizing EVs from entomopathogenic nematodes, including *H. bacteriophora*, may uncover novel virulence factors and deepen our understanding of host immune evasion strategies.

Interference with the host immune system is not restricted to EPNs. Helminth worms that have evolved together with their plant and animal hosts for a long time also use advanced processes to manipulate them [15]. Previous work has shown that helminth worms secrete EVs into the environment that can be internalized by host cells and influence host innate immunity [13]. For instance, exosomes released by *Schistosoma japonicum* adults have been shown to cause macrophage polarization and repress the Type 2 response in mice, which demonstrates the immunomodulatory activity of nematode-derived exosomes and their involvement in the infection process [16,17]. Another study also showed that *Heligmosomoides polygyrus* secreted soluble proteins and exosomes suppress IL-33 release, which further demonstrates the therapeutic potential of these molecules [18].

The conservation of fundamental biological pathways in insects makes them powerful models for dissecting innate immune signaling and function in more complex organisms [19]. For example, previous studies in the genetic model *D. melanogaster* have uncovered the molecular components that regulate the signaling pathways that lead to the expression of antimicrobial peptides (AMPs) in animals [20,21,22]. Further work has also highlighted the versatility of *D. melanogaster* for dissecting the mechanistic basis of host anti-nematode immune response [23,24,25,26,27,28,29]. For instance, exposing *D*. *melanogaster* larvae to *H*. *bacteriophora* infective juveniles induces the expression of four AMP genes. The AMP response is specific to their associated *P*. *luminescens* bacteria because nematodes devoid of *P. luminescens* (axenic nematodes) are not able to induce the expression of AMP genes [30]. Also, *H*. *bacteriophora* infection leads to the upregulation of many immune genes in *D. melanogaster* adults; however, injection of *P*. *luminescens* bacterial cells lowers immune gene expression [31]. The identification and characterization of insect defense mechanisms against EPNs substantially contributes toward devising improved tactics for the successful control of damaging insects [31]. The conservation of virulence factors between EPNs and human parasitic nematodes further provides important information on the emergence of nematode parasitism [32,33].

Here, we examined the survival response and transcriptional expression of immune-related genes in *D. melanogaster* adults following injection with *H*. *bacteriophora* extracellular vesicles and excretory-secretory products free of EVs. Our results show that *H. bacteriophora* EVs modify the expression of readout genes in Toll, Imd, Jak/Stat, Jnk, and TGF-β signaling pathways and the melanization cascade. These findings show that EPN EVs play a critical role in insect immunomodulation and strengthen the notion that EPNs produce a wide range of effector molecules to disrupt the host’s innate immune signaling during infection. This information paves the way to further exploit EPN EVs to improve the control of noxious insects.

## 2. Materials and Methods

### 2.1. Fly Stocks

The *D. melanogaster* Oregon-R line was used in all experiments. The age of the adult flies was restricted to approximately 7–10 days. Flies were maintained in an incubator (Percival, Perry, IA, USA) at 25 °C temperature with a 12 h photoperiod. Flies were kept in polystyrene narrow vials (25 × 95 mm) on 10 mL of ready-made fly food (Fly Food B recipe, Lab Express, Ann Arbor, MI, USA) supplemented with yeast.

### 2.2. Collection of Nematode Excreted–Secreted Products and Purification of Vesicles

The *H. bacteriophora* strain Az148 was obtained through injection of *Galleria mellonella* larvae with 100 infective juveniles. These were collected using a White trap, and then they were washed with sterile water and maintained at 10 °C in tap water for up to 20 days to maintain viability. Excreted–secreted (ES) products released by the infective juveniles were obtained by following the protocol described before, with slight modifications [34]. Approximately 1 million infective juveniles were disinfected and unsheathed from their cuticle via incubation in 0.01% sodium hypochlorite for 10 min, followed by two washes with sterile saline solution (0.8% NaCl). The nematodes were then transferred to Tyrode’s solution supplemented with 1% *G. mellonella* hemolymph at a concentration of 12,500 nematodes per milliliter and incubated at 23 °C with gentle agitation for 18 h to induce the parasitic stage. After induction, the nematodes were rinsed with sterile saline solution (0.8% NaCl) and subsequently passed to newly made Tyrode buffer without hemolymph. A further incubation for 3 h under the same conditions allowed for the release of ES products and EVs without host-derived contaminants.

The nematodes were then removed via filtration through filter paper, and the resulting suspension was centrifuged. The supernatant was filtered through a cellulose acetate 0.22-µm membrane filter, and the ES products were concentrated using Amicon Ultra MWCO centrifugal filters (Sigma-Aldrich, St. Louis, MO, USA) at 4 °C. The ES products were initially concentrated using 100 kDa molecular weight cut-off filters to concentrate the extracellular vesicles (EVs), which were in the retentate. The filtrate was subsequently concentrated using 10 kDa molecular weight cut-off filters to confirm the presence of ES products. The resulting fractions were stored at 4 °C until further analysis.

The pre-concentrated ES products from the 100 kDa MW filtration were subsequently loaded onto a 10 mL Sepharose CL-2B cross-linked agarose gel filtration column (Cytiva, Maharashtra, India) integrated with an ÄKTA Pure chromatograph (GE Healthcare Life Sciences, Bangalore, India). Elution was carried out using 50 mM of phosphate buffer (PBS; pH 7.2, 0.8% NaCl). The EV fractions were collected based on the 280 nm UV absorbance. The collected fractions were then concentrated with an Amicon Ultra-15 10 kDa molecular weight cut-off filter before washing with 30 mL of PBS and concentrating further to a final volume of 100 μL. The concentrated and purified EVs were kept at −80 °C for subsequent downstream analyses.

### 2.3. Nanoparticle Tracking Analysis

Analysis of nanoparticle tracking was conducted as a service by Paralab SA (Valbom, Portugal) deploying a NanoSight NS300 (Malvern Instruments, Malvern, UK) supplied with a blue 488 nm laser and NTA software version 3.2 (Dev Build 3.2.16). Diluted samples were prepared in PBS and analyzed according to Paralab’s standard protocol. Measurements were conducted at a temperature of 24.7 °C with a camera level set to 9, a slider shutter of 607, and a slider gain of 15. Each sample was measured by recording five 30 s videos with 18.5 frames per second (FPS) and a total of 463 frames captured. The analysis was performed with a syringe pump speed of 15. Analysis parameters were kept consistent across all measurements, with the detection limit set to 3, automatic blur size, and a maximum jump distance ranging between 16.4 and 23.0 pixels. Measurements were also conducted in light scatter mode using the same camera settings.

### 2.4. Preparation of Dead Nematodes

*H. bacteriophora* infective juveniles (approximately 200,000 nematodes) were removed from a tissue culture flask and heated at 60 °C for 15 min to kill them and inactivate any remaining bacteria. The infective juveniles were centrifuged in 1.5 mL Eppendorf tube for 30 s at 1300× *g* and then homogenized in 1 mL of sterile PBS using autoclaved reusable plastic pestles and a handheld homogenizer (MilliporeSigma, Burlington, MA, USA). The nematode homogenate was stored at 4 °C until use.

### 2.5. Fly Survival Experiments

*D. melanogaster* 7–10-day-old adult female flies of the Oregon-R line were knocked out with carbon dioxide and then intrathoracically injected with 69.0 nL of sterile PBS (negative control), homogenized *H. bacteriophora* (4 μg/μL), nematode excreted–secreted products, or nematode EVs corresponding to 100 infective juveniles using a Nanoject III Programable Nanoliter Injector (Drummond Scientific, Broomall, PA, USA). All fly injections were performed in the morning. The injected flies were transferred to ready-made fly food supplemented with yeast and kept at 25 °C and 12 h light photoperiod. Fly survival results were recorded daily and up to 8 days after injection. Three separate experiments were performed on different days, each containing five replicates with 10 adult female flies per replicate.

### 2.6. Fly Gene Transcriptional Expression

*D. melanogaster* 7–10 days old Oregon-R adult flies were injected as described above. Injected flies were harvested 6 and 24 h after injection and placed at −80 °C. Extraction of total RNA from each sample involved homogenization in TRIzol reagent (Thermo Fisher Scientific, Rockville, MD, USA) from two male and two female flies. Overall, three replicates for each time point were performed. RNA concentrations were normalized to 1000 ng/μL, and cDNA templates were synthesized using the High-Capacity cDNA Reverse Transcription Kit (Applied Biosystems, Carlsbad, CA, USA). Gene expression experiments were carried out on a C1000 Thermal Cycler CFX96 Real-Time System (Bio-Rad, Philadelphia, PA, USA). The cycle conditions involved 95 °C for 2 min and 40 repeats of 95 °C for 15 s, 61 °C for 30 s, 95 °C for 15 s, 65 °C for 5 s, and 95 °C for 5 s. Each qPCR reaction had a final volume of 20 μL consisting of 10 μL of GreenLink No-ROX qPCR Mix (Biolink Life Sciences, Cary, NC, USA), 40 ng of each cDNA sample, the two primers (forward and reverse) at a working concentration of 200 nM, and 1.2 μL of sterile deionized water. Primers (sequences listed in Table 1) for the tested genes were designed with the Primer3 PCR primer design tool, and they were purchased from Azenta Life Sciences (Burlington, MA, USA). Gene expression results from the qRT-PCR assays were processed based on the 2^−ΔΔCT^ method [35,36]. The dCt numbers were calculated from three experiments with two technical repetitions for each sample. The ddCt values, together with standard error bars, are plotted in the figures.

### 2.7. Statistical Analysis

Statistical analysis of the data was completed with GraphPad Prism 5 software. Results from the fly survival assays were analyzed using the log-rank (Mantel-Cox) method with 95% CIs. Gene expression results were analyzed and correlated with a one-way ANOVA and Bonferroni multiple comparisons test to detect differences between the various experimental conditions. The relative expression in the same gene at 6 and 24 h was compared using a *t*-test. All statistical analyses were performed on data collected through three independent experiments.

## 3. Results

### 3.1. Characterization of Exosomal Vesicles from H. bacteriophora

**Figure 1:**
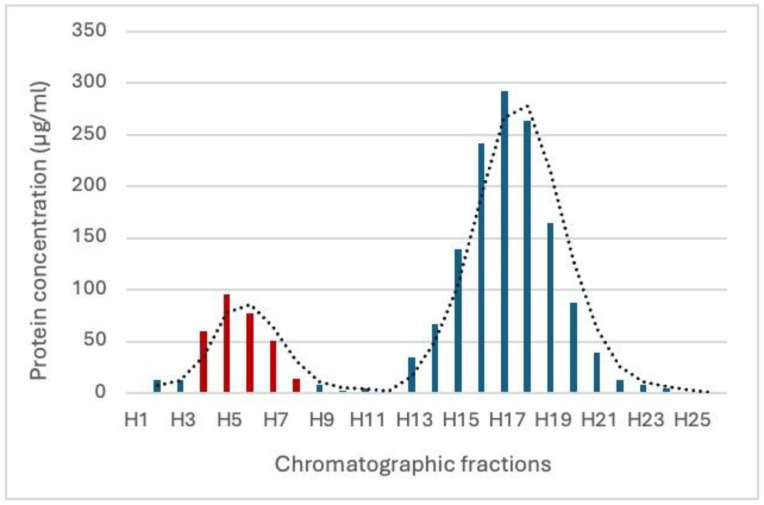
Chromatogram of concentrated ES products from the 100 kDa MW loaded onto a Sepharose CL-2B gel filtration column. The first peak (red bars) observed between fractions H3 and H7 represents the EV-rich fraction, which was collected for downstream NTA analysis. The second peak (blue bars), detected between fractions H13 and H21, corresponds to soluble proteins without vesicles. The dotted line represents the protein concentration profile across the chromatographic fractions.

**Figure 2:**
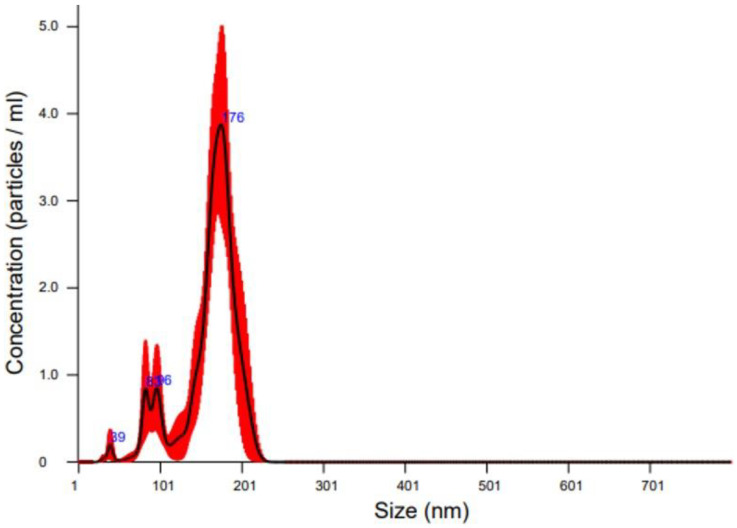
Nanoparticle Tracking Analysis of purified extracellular vesicles from *H. bacteriophora*, strain Az148. The graph shows the size distribution and concentration of EVs. The analysis was performed using a NanoSight NS300 with NTA software version 3.2 and a blue 488 nm laser. The sample was measured at 24.7 °C with a camera level of 9, gain of 15, and a syringe pump speed of 15.

The chromatogram of the pre-concentrated ES products from the 100 kDa MW filtration revealed two distinct peaks, corresponding to different fractions eluted from the Sepharose CL-2B column. The first elution peak, observed between fractions 3 and 7 mL, corresponds to the EV-rich fraction, which was confirmed downstream using NTA analysis (Figure 1). The second peak, detected between fractions H13 and H21, primarily consists of soluble protein components without vesicles, representing free proteins released during the nematode secretion process. The NTA analysis revealed that the purified fraction likely contains intact vesicles, as indicated by the early elution characteristic of larger particles. The particle concentration in the purified EV fraction ranged from 1.54 × 10^8^ to 1.72 × 10^7^ particles/mL. The size distribution showed a predominant population of vesicles with an average diameter of 149.0 nm and a modal size of 175.2 nm, indicating that most EVs fall within the typical exosome size range and suggesting successful isolation (Figure 2). Curiously, a secondary population of vesicles, representing approximately 10% of the total vesicle population, was detected with an average size of 89 nm. This smaller population may reflect a heterogeneous vesicle population or smaller EV subtypes.

### 3.2. H. bacteriophora EVs Are Lethal to D. melanogaster Adult Flies

**Figure 3:**
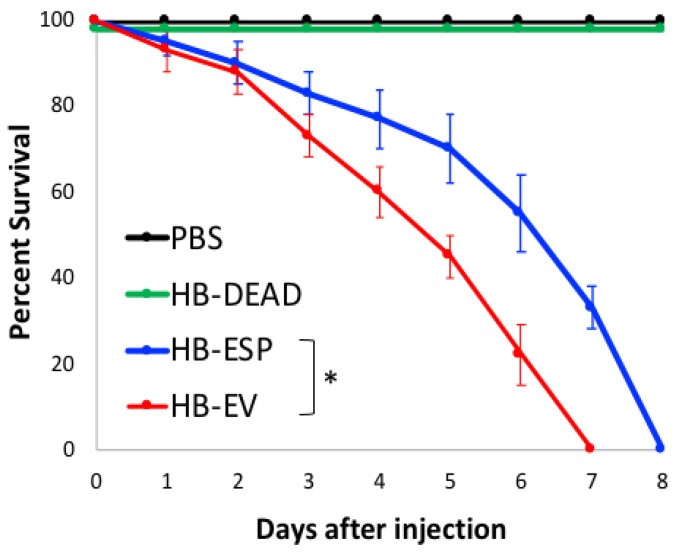
Fly survival response to entomopathogenic nematode EVs. *D. melanogaster* Oregon-R adult flies were injected in the thorax with phosphate-buffered saline (PBS), homogenized *H. bacteriophora* (HB-DEAD), *H. bacteriophora* excreted–secreted products (HB-ESP), and *H. bacteriophora* EVs (HB-EV). Fly survival was monitored up to 8 days following injection. Fly experiments were repeated three times with 60 *D. melanogaster* adults (5–10 days old) per experimental treatment. The asterisk indicates a value that is significantly different (Mantel–Cox, df = 1, * *p* < 0.05). Error bars denote standard errors.

To test the lethality of EPN EVs to insects, we injected *D. melanogaster* wild-type adults with EVs isolated from *H. bacteriophora* infective juveniles and monitored the survival of flies over time (Figure 3). As controls, we injected flies with PBS (vehicle control), homogenized *H. bacteriophora* infective juveniles (nematode negative control, HB-DEAD), and *H. bacteriophora* excreted–secreted products (nematode positive control, HB-ESP).

We found no fly mortality upon injection of PBS and HB-DEAD into flies, which survived these challenges for the duration of the experiment (i.e., 8 days post-injection). Also, as we showed before [11], we found that HB-ESP was lethal to flies, which succumbed 7 days after injection. Interestingly, we further observed that HB-EV killed flies at a significantly higher rate compared to HB-ESP (Mantel-Cox, df = 1, * *p* < 0.05), as all flies died 7 days after the challenge.

These results indicate that HB-EV possesses pathogenic properties for insects and that *H. bacteriophora* nematodes employ various effectors to harm insect hosts following invasion.

### 3.3. H. bacteriophora EVs Suppress the Expression of Attacin and Cecropin in Adult D. melanogaster

To explore the effect of *H. bacteriophora* EVs on the *D. melanogaster* Toll and Imd signaling activity, we injected these nematode components into adult flies and quantified the transcriptional expression of read-out genes for each pathway.

**Figure 4:**
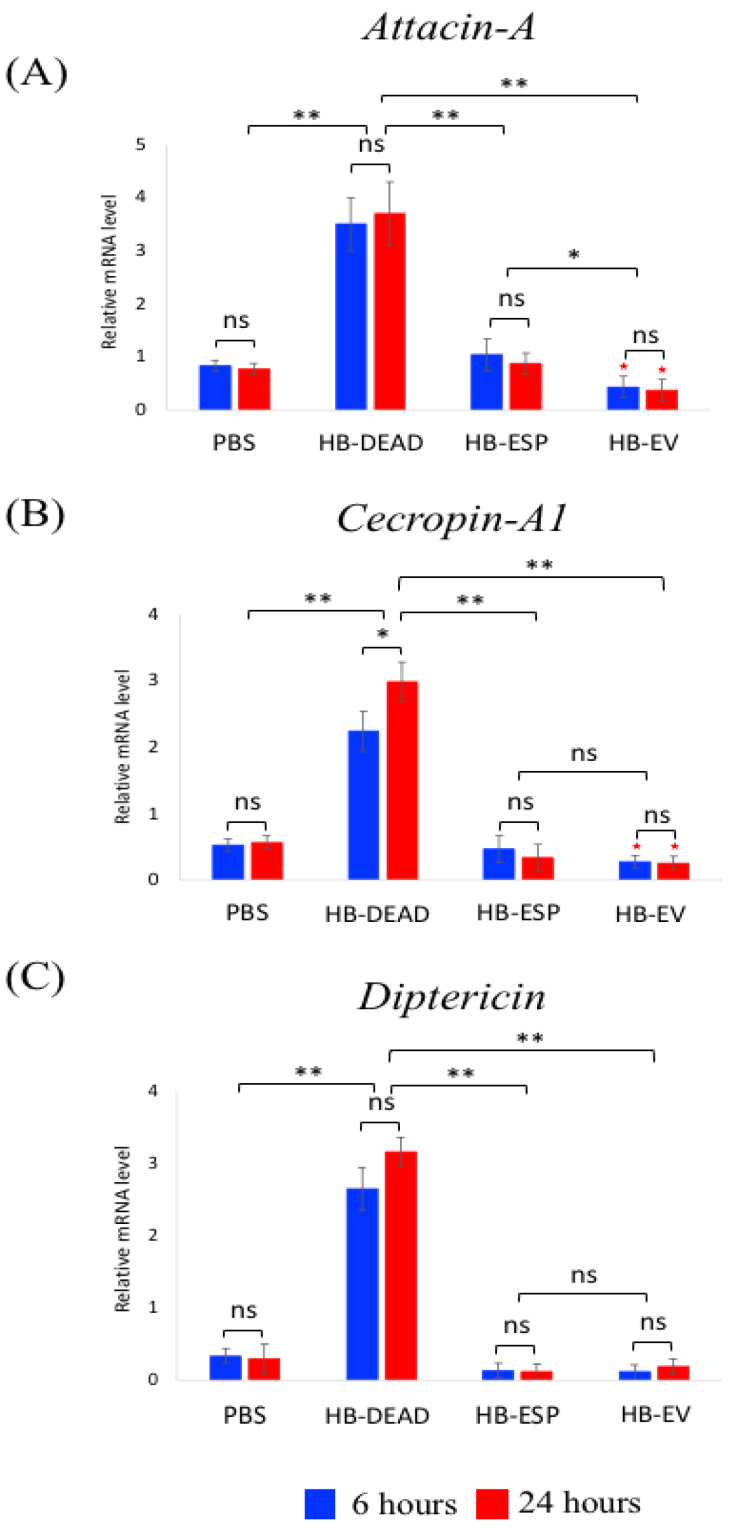
Transcriptional expression of immune deficiency (Imd) pathway genes. *D. melanogaster* Oregon-R adult flies were intrathoracically injected with phosphate-buffered saline (PBS), *H. bacteriophora* axenic homogenized nematodes (HB-DEAD), *H. bacteriophora* excreted–secreted products (HB-ESP), and *H. bacteriophora* EVs (HB-EV). Expression levels of (**A**) *Attacin*, (**B**) *Cecropin*, and (**C**) *Diptericin* were assessed at 6 and 24 h after injection. Levels of mRNA are presented as the relative abundance of transcripts normalized to *RpL32* and are expressed as a ratio compared to flies injected with PBS alone (negative controls). Values are the means from three independent experiments, and error bars are standard deviations. Asterisks (*) indicate a value that is significantly different (one-way ANOVA with a Tukey post hoc test for multiple comparisons). **, *p* < 0.01; *, *p* < 0.05; ns, non-significant differences. Red stars (★, *p* < 0.05) indicate significantly lower gene expression in HB-EV treatments compared to the PBS control. Comparison between relative gene expression at 6 and 24 h was performed with a *t*-test.

We found that challenging *D. melanogaster* adult flies with the nematode EVs decreased the transcriptional expression of *Attacin-A* and *Cecropin-A1* (*p* = 0.038 and *p* = 0.041, respectively), but not *Diptericin* (*p* = 0.17), compared to the PBS control (Figure 4). The expression of *Attacin* (Figure 4A), *Cecropin* (Figure 4B), and *Diptericin* (Figure 4C) increased significantly upon the injection of homogenized *H. bacteriophora* (HB-DEAD) compared to injection with PBS (control) (*p* = 0.0045, *p* = 0.0071, and *p* = 0.0023, respectively). Injection of *H. bacteriophora* excreted–secreted products (HB-ESP) upregulated *Attacin*, *Cecropin*, and *Diptericin* to similar levels as injection with PBS, which was significantly lower compared to injection with dead nematodes (*p* = 0.0085, *p* = 0.0074, and *p* = 0.0056, respectively) (Figure 4A–C). We found no statistically significant variation in the expression of any of the three genes between the two time points (*p* > 0.05).

**Figure 5:**
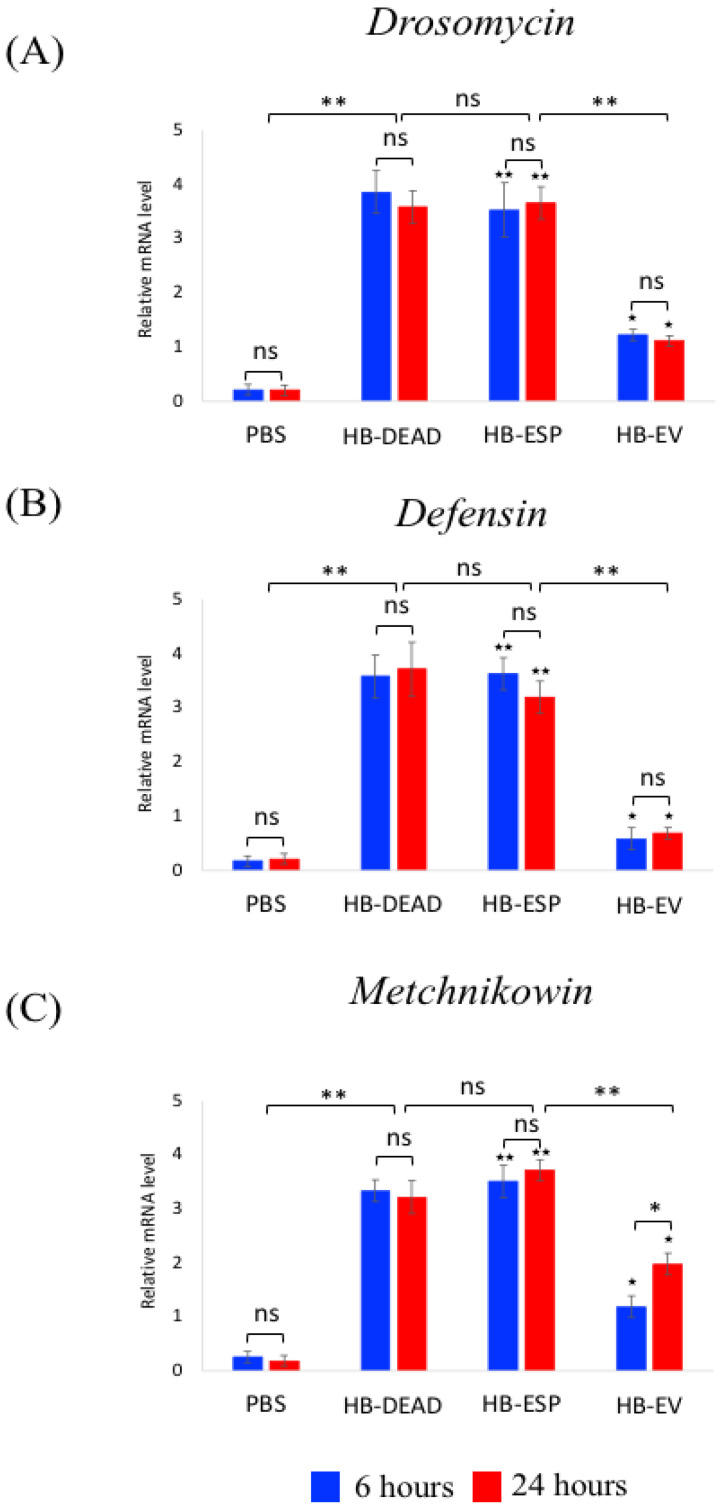
Transcriptional expression of Toll pathway genes. *D. melanogaster* Oregon-R adult flies were intrathoracically injected with phosphate-buffered saline (PBS), *H. bacteriophora* axenic homogenized nematodes (HB-DEAD), *H. bacteriophora* excreted–secreted products (HB-ESP), and *H. bacteriophora* EVs (HB-EV). Expression levels of (**A**) *Drosomycin*, (**B**) *Defensin*, and (**C**) *Metchnikowin* were assessed at 6 and 24 h after injection. Levels of mRNA are presented as the relative abundance of transcripts normalized to *RpL32*. Values are the means from three independent experiments, and error bars are standard deviations. Asterisks indicate a value that is significantly different (one-way ANOVA with a Tukey post hoc test for multiple comparisons). **, *p* < 0.01; *, *p* < 0.05; ns, non-significant differences. Black stars (★★, *p* < 0.01; ★, *p* < 0.05) indicate significantly higher gene expression in either HB-ESP or HB-EV treatments compared to the PBS control. Comparison between relative gene expression at 6 and 24 h was performed with a *t*-test.

For Toll pathway signaling activity, we quantified the transcriptional expression of three readout genes encoding antimicrobial peptides (Figure 5). As with the Imd regulated genes, we found that *Drosomycin* (Figure 5A), *Defensin* (Figure 5B), and *Metchnikowin* (Figure 5C) were upregulated upon injection with homogenized *H. bacteriophora* (HB-DEAD), and this upregulation was significantly higher compared to the PBS treatment (*p* = 0.0024, *p* = 0.0041 and *p* = 0.0055, respectively). Injection with *H. bacteriophora* excreted–secreted products (HB-ESP) also significantly upregulated *Drosomycin*, *Defensin*, and *Metchnikowin* compared to the PBS control (*p* = 0.0033, *p* = 0.0039, and *p* = 0.0028, respectively), and their expression did not differ compared to the HB-DEAD treatment. Injection of *H. bacteriophora* EVs (HB-EV) upregulated the three antimicrobial peptide genes at significantly higher level compared to the PBS treatment (*p* = 0.047, *p* = 0.026 and *p* = 0.038, respectively) but at statistically lower level than the other two treatments involving nematode components (*p* = 0.0030, *p* = 0.0043 and *p* = 0.0071, respectively). Also, the transcriptional expression of *Metchnikowin* at 6 h was lower compared to 24 h (*p* = 0.038) (Figure 5C), and there were no other statistically significant changes in gene expression between the two time points for any of the three genes (*p* > 0.05).

These results indicate that *H. bacteriophora* EVs interfere with Imd and Toll signaling activity, as shown by the downregulation of the antimicrobial peptide-encoding genes *Attacin-A* and *Cecropin-A1*.

### 3.4. H. bacteriophora EVs Moderately Activate the JNK and Jak/Stat Signaling Pathways

**Figure 6:**
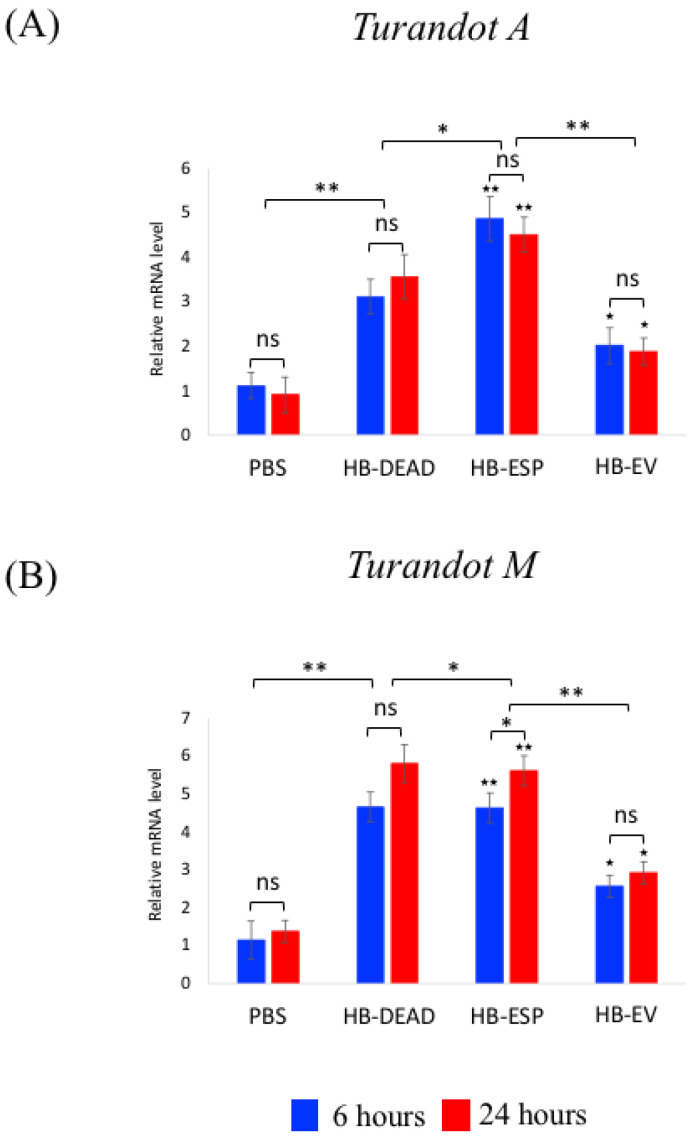
Transcriptional expression of Jak/Stat pathway genes. *D. melanogaster* Oregon-R adult flies were intrathoracically injected with phosphate-buffered saline (PBS), *H. bacteriophora* axenic homogenized nematodes (HB-DEAD), *H. bacteriophora* excreted–secreted products (HB-ESP), and *H. bacteriophora* EVs (HB-EV). Expression levels of (**A**) *Turandot A* and (**B**) *Turandot M* were estimated at 6 and 24 h post-injection. Levels of mRNA are shown as the relative abundance of transcripts normalized to *RpL32* and are expressed as a ratio compared to flies injected with PBS only (negative controls). Values are the means from three independent experiments, and error bars are standard deviations. Asterisks indicate a value that is significantly different (one-way ANOVA with a Tukey post hoc test for multiple comparisons). **, *p* < 0.01; *, *p* < 0.05; ns, non-significant differences. Black stars (★★, *p* < 0.01; ★, *p* < 0.05) indicate significantly higher gene expression in either HB-ESP or HB-EV treatments compared to the PBS control. Comparison between relative gene expression at 6 and 24 h was performed with a *t*-test.

Next, we investigated changes in Jak/Stat pathway activity by estimating the transcriptional induction of two Turandot genes (*Turandot A* and *Turandot M*), which are normally induced by stress conditions [37] (Figure 6). We found that both *Turandot A* and *Turandot M* were significantly upregulated in flies that had been previously injected with either homogenized *H. bacteriophora* axenic nematodes (HB-DEAD) (*p* = 0.0082 and *p* = 0.0066, respectively) or excreted–secreted products (HB-ESP) (*p* = 0.0059 and *p* = 0.0036, respectively) compared to those injected with PBS (Figure 6A,B). In fact, *Turandot A* expression in the HB-ESP treatment was similar at both time points (*p* = 0.067); however, the expression of *Turandot M* was significantly higher at 24 h compared to the 6 h time point (*p* = 0.043). In flies injected with *H. bacteriophora* EVs (HB-EV), the expression of *Turandot A* and *Turandot M* was substantially upregulated compared to the PBS control (*p* = 0.069 and *p* = 0.072, respectively), but their levels were significantly lower compared to the HB-ESP treatment (*p* = 0.0054 and *p* = 0.0048, respectively). No statistically significant differences between the two time points were noted (*p* > 0.05) (Figure 6A,B).

**Figure 7:**
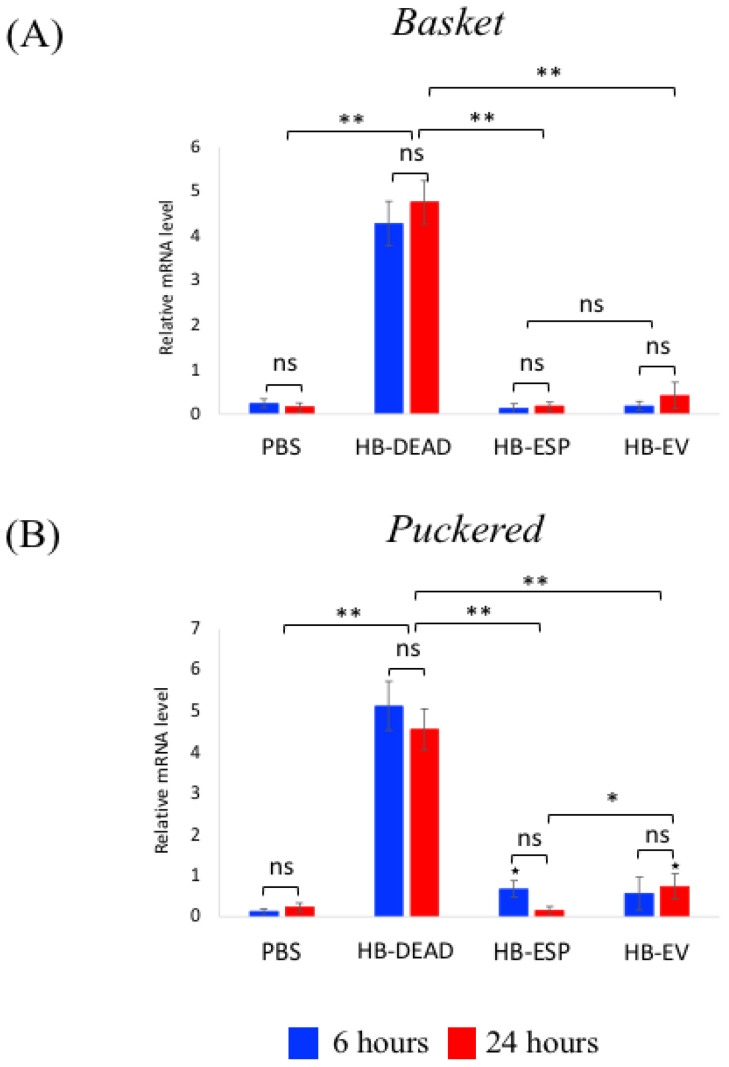
Transcriptional expression of Jnk pathway genes. *D. melanogaster* Oregon-R adult flies were intrathoracically injected with phosphate-buffered saline (PBS), *H. bacteriophora* axenic homogenized nematodes (HB-DEAD), *H. bacteriophora* excreted–secreted products (HB-ESP), and *H. bacteriophora* EVs (HB-EV). Expression levels of (**A**) *Basket* and (**B**) *Puckered* were assessed at 6 and 24 h after injection. Levels of mRNA are presented as the relative abundance of transcripts normalized to *RpL32*. Values are the means from three independent experiments, and error bars are standard deviations. Asterisks indicate a value that is significantly different (one-way ANOVA with a Tukey post hoc test for multiple comparisons). **, *p* < 0.01; *, *p* < 0.05; ns, non-significant differences. Black stars (★, *p* < 0.05) indicate significantly higher gene expression in either HB-ESP or HB-EV treatments compared to the PBS control. Comparison between relative gene expression at 6 and 24 h was performed with a *t*-test.

For Jnk pathway signaling activity, we tested the expression of the readout genes *Basket* and *Puckered* (Figure 7). We found that both genes were substantially upregulated in response to dead *H. bacteriophora* (HB-DEAD) compared to the PBS control (*p* = 0.0021 and *p* = 0.0018, respectively), with no statistically significant changes between the two time points (*p* > 0.05) (Figure 7A,B). In contrast, *Basket* and *Puckered* were induced at low levels after injection with either *H. bacteriophora* excreted–secreted products (HB-ESP) or EVs (HB-EV). *Basket* and *Puckered* gene expression levels were significantly higher in HB-ESP injected flies and HB-EV injected flies at 6 and 24 h compared to those observed in the PBS control treatment (*p* = 0.031 and *p* = 0.0024, respectively) (Figure 7A,B). Although no statistically significant changes in *Basket* expression levels were noted between the two time points within the HB-ESP and HB-EV treatments, the expression of *Puckered* in HB-ESP-treated flies was significantly lower at 24 h post-injection compared to the HB-EV-treated individuals (*p* = 0.037) (Figure 7B).

These findings suggest that *H. bacteriophora* EVs induce Jak/Stat and Jnk signaling in adult flies at a moderate level.

### 3.5. H. bacteriophora EVs Induce the TGF-β Signaling and Downregulate PPO3 Gene Expression in D. melanogaster Adults

**Figure 8:**
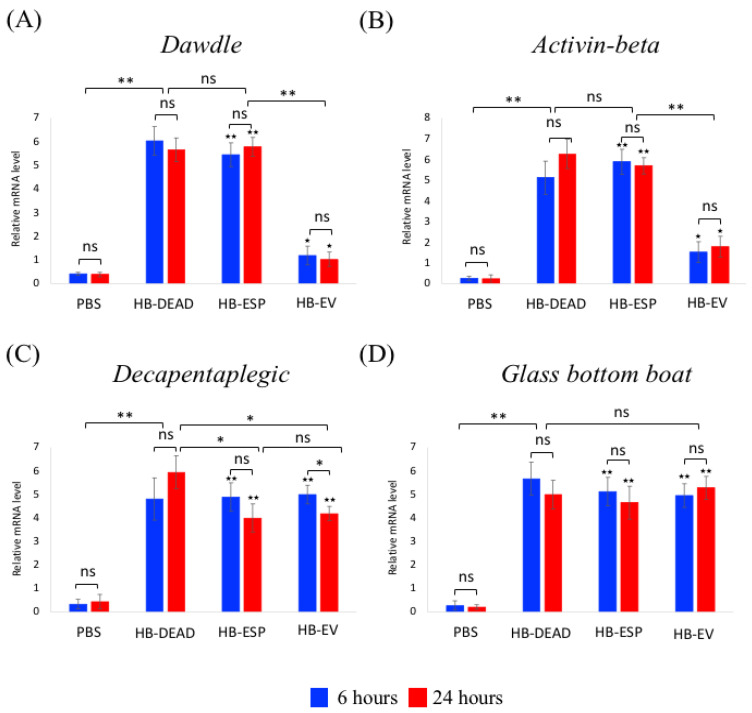
Transcriptional expression of TGF-β pathway genes. *D. melanogaster* Oregon-R adult flies were injected intrathoracically with phosphate-buffered saline (PBS), *H. bacteriophora* axenic homogenized nematodes (HB-DEAD), *H. bacteriophora* excreted–secreted products (HB-ESP), and *H. bacteriophora* EVs (HB-EV). Expression levels of (**A**) *Dawdle*, (**B**) *Activin-β* (Activin branch), (**C**) *Decapentaplegic*, and (**D**) *Glass bottom boat* (Bone Morphogenetic Protein branch) were assessed at 6 and 24 h after injection. Levels of mRNA are presented as the relative abundance of transcripts normalized to *RpL32*. Values are the means from three independent experiments, and error bars are standard deviations. Asterisks indicate a value that is significantly different (one-way ANOVA with a Tukey post hoc test for multiple comparisons). **, *p* < 0.01; *, *p* < 0.05; ns, non-significant differences. Black stars (★★, *p* < 0.01; ★, *p* < 0.05) indicate significantly higher gene expression in either HB-ESP or HB-EV treatments compared to the PBS control. Comparison between relative gene expression at 6 and 24 h was performed with a *t*-test.

The two signaling branches of the TGF-β pathway, Bone Morphogenetic Protein (BMP) and Activin, have previously been implicated in the *D. melanogaster* response to *Heterorhabditis* sp. infection [38,39,40,41,42,43,44]. Here, we examined the transcriptional expression of representative genes in both TGF-β branches in wild-type flies inoculated with *H. bacteriophora* EVs (Figure 8). We found that both *Dawdle* and *Activin-β* were significantly upregulated via the injection of homogenized *H. bacteriophora* axenic nematodes (HB-DEAD) (*p* = 0.0067 and *p* = 0.0051, respectively) or excreted–secreted products (HB-ESP) (*p* = 0.0047 and *p* = 0.0038, respectively) compared to the PBS treatment, and there was no statistical significant changes between the two experimental conditions (*p* = 0.71 and *p* = 0.59, respectively) (Figure 8A,B). Both genes were significantly upregulated in response to *H. bacteriophora* EVs (HB-EV) compared to the PBS control (*p* = 0.088 and *p* = 0.0067, respectively), but their induction was significantly lower than the HB-ESP treatments (*p* = 0.0056 and *p* = 0.0053, respectively) (Figure 8A,B). No statistically significant differences in *Dawdle* and *Activin-β* expression were noted between the two time points for any of the experimental conditions (*p* > 0.05). For the BMP branch ligands, we found that the mRNA levels of *Decapentaplegic* (*Dpp*) and *Glass bottom boat* (*Gbb*) were substantially higher in flies injected with any of the *H. bacteriophora* preparations than in PBS-injected individuals (*Dpp*: *p* = 0.0077, *p* = 0.0065, and *p* = 0.0062, respectively; *Gbb*: *p* = 0.0058, *p* = 0.0067, and *p* = 0.0071, respectively) (Figure 8C,D). The expression of *Dpp* at 24 h was considerably lower in the HB-EV injected flies compared to the HB-DEAD treated individuals (*p* = 0.083) but statistically similar to the *Dpp* expression in HB-ESP injected flies (*p* = 0.066) (Figure 8C). Also, the expression of *Dpp* at 24 h post-injection of HB-EV was statistically lower compared to the 6 h time point (*p* = 0.041). No statistically significant changes in *Gbb* expression were found between the 6 and 24 h time points for any of the injection treatments (*p* > 0.05) (Figure 8D).

**Figure 9:**
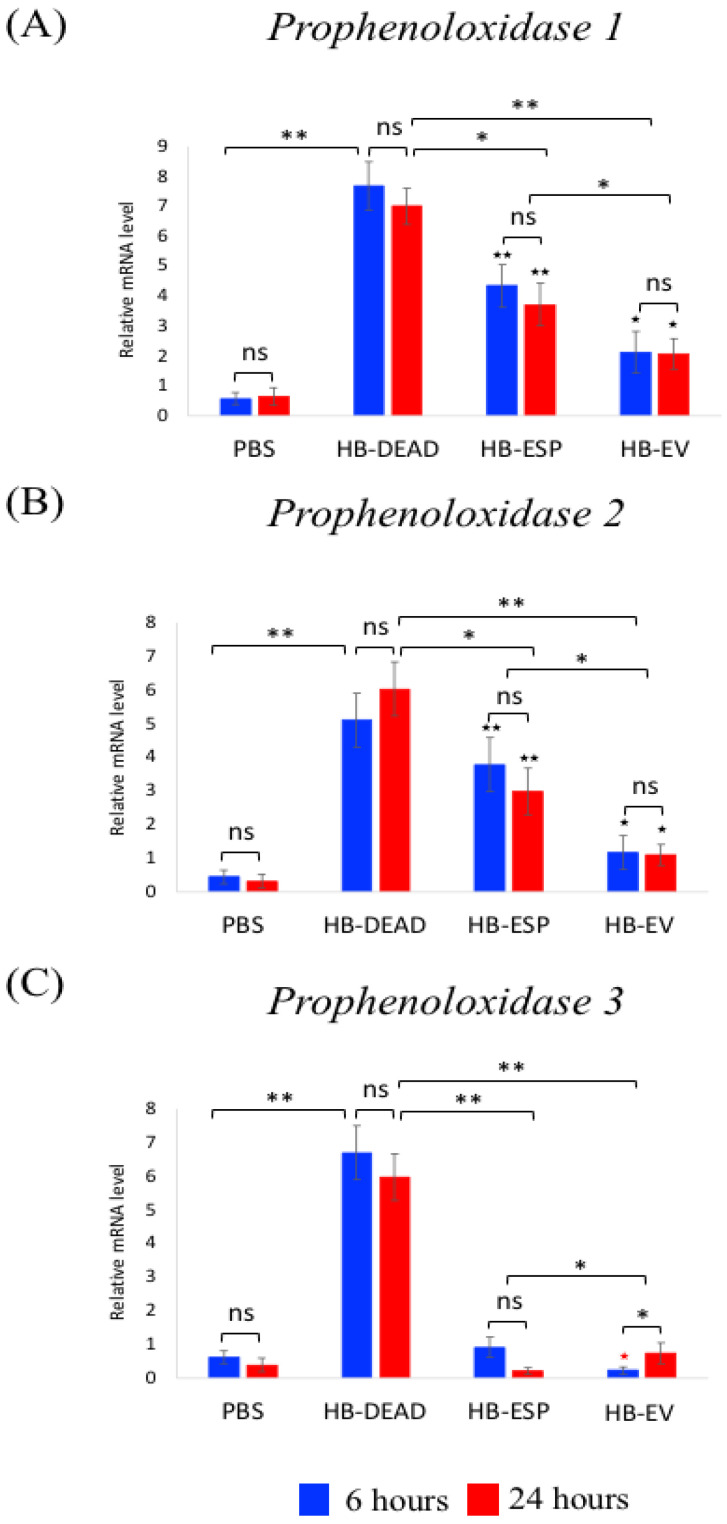
Transcriptional expression of Prophenoloxidase pathway genes. *D. melanogaster* Oregon-R adult flies were intrathoracically injected with phosphate-buffered saline (PBS), *H. bacteriophora* axenic homogenized nematodes (HB-DEAD), *H. bacteriophora* excreted–secreted products (HB-ESP), and *H. bacteriophora* EVs (HB-EV). Expression levels of (**A**) *Prophenoloxidase 1*, (**B**) *Prophenoloxidase 2*, and (**C**) *Prophenoloxidase 3* were assessed at 6 and 24 h after injection. Levels of mRNA are presented as the relative abundance of transcripts normalized to *RpL32*. Values are the means from three independent experiments, and error bars are standard deviations. Asterisks indicate a value that is significantly different (one-way ANOVA with a Tukey post hoc test for multiple comparisons). **, *p* < 0.01; *, *p* < 0.05; ns, non-significant differences. Black asterisks (★) indicate significantly increased gene expression in either HB-ESP or HB-EV treatments compared to the PBS control. Black stars (★★, *p* < 0.01; ★, *p* < 0.05) indicate significantly higher gene expression in either HB-ESP or HB-EV treatments compared to the PBS control. Red star (★, *p* < 0.05) indicates significantly lower gene expression in the HB-EV treatment compared to the PBS control. Comparison between relative gene expression at 6 and 24 h was performed with a *t*-test.

To assess the influence of EPN EVs on the adult fly melanization cascade, we quantified the transcriptional expression of three prophenoloxidase (PPO)-encoding genes (Figure 9). We found that the *PPO1* and *PPO2* gene expression levels were highly upregulated upon injection of dead axenic *H. bacteriophora* (HB-DEAD) (*p* = 0.0022 and *p* = 0.0035, respectively), they were moderately upregulated upon injection of *H. bacteriophora* excreted–secreted products (HB-ESP) (*p* = 0.0044 and *p* = 0.0059, respectively), and they were slightly upregulated upon injection of *H. bacteriophora* EVs (HB-EV) (*p* = 0.044 and *p* = 0.0027, respectively) compared to PBS controls (Figure 9A,B). The upregulation of *PPO1* and *PPO2* was significantly lower in the HB-EV injected flies compared to HB-ESP injected flies (*p* = 0.0056 and *p* = 0.0051, respectively). There were no significant changes in *PPO1* and *PPO2* expression between the two time points (*p* > 0.05) (Figure 9A,B). Interestingly, *PPO3* gene expression was not induced after HB-ESP injection compared to PBS treatment (*p* = 0.068). Finally, *PPO3* gene expression was significantly lower in HB-EV injected flies at the 6 h time point compared to the PBS control (*p* = 0.0074) and at the 24 h time point compared to the HB-EV-treated individuals (*p* = 0.039) (Figure 9C).

These results imply that *H. bacteriophora* EVs induce the expression of TGF-β signaling pathway genes and downregulate the expression of *PPO3* in adult *D. melanogaster*.

## 4. Discussion

**Figure 10:**
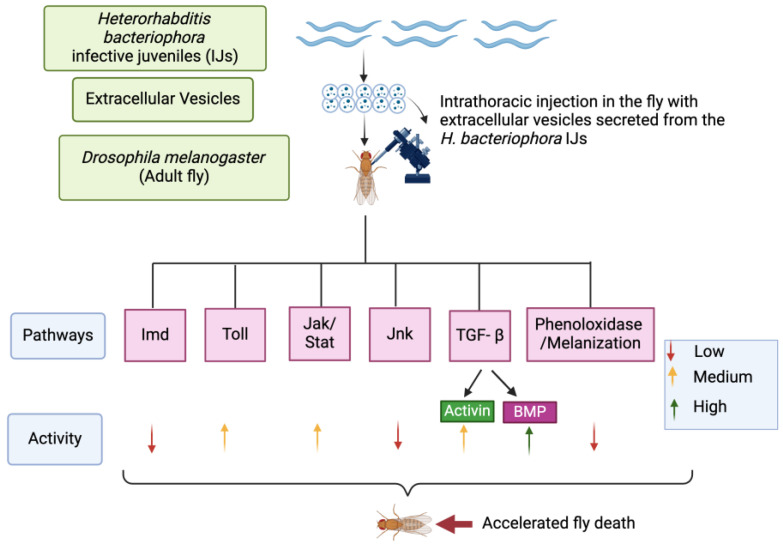
Effect of *H. bacteriophora* extracellular vesicles (EVs) on the *D. melanogaster* immune response and survival. Injection of EVs from *H. bacteriophora* infective juveniles into wild-type adult flies (black arrows) interferes with several innate immune signaling pathways and reduces fly survival.

Entomopathogenic nematodes constitute an integral component of biological control strategies against harmful insects [45,46]. The pathogenic efficiency of *H. bacteriophora* nematodes in eliminating insects is due to their ability to release infection factors and effector molecules together with their symbiotic bacteria *P. luminescens* during the early stages of infection [3,47]. These processes are better analyzed in model insects that have a well-described innate immune system. Here, we adopted *D. melanogaster* to investigate the effect of EVs from *H. bacteriophora* infective juveniles on the *D. melanogaster* immune signaling activity [48,49]. Our findings suggest that *H. bacteriophora* EVs do not strongly activate the immune response signaling in *D. melanogaster* and may instead contribute to immune modulation (Figure 10). This novel information is critical for interpreting the molecular foundation of entomopathogenic nematode infection. Also, it reveals how these parasites disrupt the activity of highly conserved signaling pathways in order to interfere with the insect’s innate immune system.

*H. bacteriophora* nematodes utilize an assortment of virulence factors that contribute to insect death. Previous work has determined that activated *H. bacteriophora* excreted–secreted products (ESP) are virulent to *D. melanogaster* flies, as injection of approximately 414 infective juvenile equivalents resulted in substantial fly mortality over a period of 6 h [11]. In contrast, wild-type flies challenged with a recombinant *H. bacteriophora* ecdysteroid glycosyltransferase, which was previously detected in the hemolymph-activated transcriptome of this EPN, survived comparably to buffer-injected individuals [50]. Two other *H. bacteriophora* virulence factors, a putative lysozyme and a serine carboxypeptidase, when injected into wild-type adult flies in recombinant form, caused slight mortality, which was substantially increased upon coinjection with the symbiotic bacteria *P. luminescens* [51]. Similarly, wild-type flies were able to survive the injection of an *H. bacteriophora* recombinant serine carboxypeptidase [52]. Here, we demonstrate that *H. bacteriophora* EVs are capable of killing wild-type flies within a week of injection. This finding adds another weapon to the arsenal of *H. bacteriophora* and implies the immunomodulatory capacity of EPN EVs in *D. melanogaster* and potentially in other insects.

The immunomodulatory capacity of *H. bacteriophora* EVs is first demonstrated by the low induction of the three Imd pathway-regulated antimicrobial peptide-encoding genes, *Attacin*, *Cecropin,* and *Diptericin*. We previously found that *H. bacteriophora* ESPs suppress the signaling level of the Imd pathway [11]. Here, we extend these findings by showing the low expression of the three Imd genes in HB-ESP-injected flies. The effect of both *H. bacteriophora* EVs and excreted–secreted products on Imd signaling activity suggests the potential involvement of this pathway in opposing EPN infection in *D. melanogaster*. Curiously, we observed a more pronounced decrease in *Attacin* expression by EVs compared to ESP, whereas *Cecropin* and *Diptericin* were also downregulated but to a lesser extent. This suggests that different *H. bacteriophora* infection factors may selectively target specific antimicrobial peptides. Previously, we showed that *H. bacteriophora* ESPs downregulate the expression of *Diptericin* [11]; therefore, it is possible that different EPN infection factors target specific antimicrobial peptide genes to disarm the fly immune system. Also, the low expression level of the three Toll pathway antimicrobial peptide-encoding genes *Drosomycin*, *Defensin,* and *Metchnikowin* by *H. bacteriophora* EVs, but not by ESPs, further suggests that EPN EVs have the capacity to interfere with the two NF-κB pathways in *D. melanogaster*.

Testing the Jak/Stat signaling activity revealed that injection of *H. bacteriophora* excreted–secreted products strongly induced the expression of both *Turandot A* and *Turandot M*, which was markedly higher compared to the gene expression levels upon injection with *H. bacteriophiora* EVs. Because the *Tot* genes encode small peptides that are expressed in response to various stresses [53], these findings imply that EPN EVs, but not excreted–secreted products, can reduce the stress response of the fly during infection. Importantly, both Jnk pathway readout genes Basket and Puckered were marginally induced by *H. bacteriophora* excreted–secreted products as well as EVs. This gene expression trend was similar to the pattern observed for the Imd pathway readout genes. Of note, the Imd pathway induces the Jnk pathway through activated TGF-β activated kinase 1 (Tak1) in *D. melanogaster* [22,54]; therefore, these EPN molecules seem to specifically target both signaling pathways during infection of the adult fly. The significance of this EPN infection strategy remains to be investigated in future studies.

*D. melanogaster* deploys the Bone Morphogenetic Protein (BMP) and Activin branches of the TGF-β signaling pathway [55]. Previously, we revealed the role of both BMP and Activin signaling branches in the host immunity to nematode parasites. We established that Activin signaling promotes anti-nematode immunity in *D. melanogster* because inactivation of *Daw* reduces fly survival to infection by *Heterorhabditis* EPNs and increases the persistence of the nematodes in the mutants [44]. Also, mutation of *Mad* or *Dpp* improves fly survival and elevates the antimicrobial peptide gene expression in response to sterile injury or nematode attack, respectively, but not in response to bacterial infection [43]. Interestingly, the BMP pathway members *Scw* and *Sax*, together with the Activin pathway component *Babo*, were significantly induced upon *H. gerrardi* infection, which results in the upregulation of the intracellular regulator *Mad* [42]. Also, we established the essential role of NF-κB transcription factors for triggering the TGF-β signaling in *D. melanogaster*, which occurs extracellularly and is restricted to *H. gerrardi* infection [41], and further showed that *H. gerrardi*-infected *Daw* mutants express high levels of *DUOX* but low levels of phenoloxidase activity [40]. Therefore, here, we aim to expand these findings by linking TGF-β signaling to immune and metabolic function against a PN infection factor. More recently, we have found that Daw modulates the *D. melanogaster* immune response and lipid metabolism against a serine carboxypeptidase that is produced by *H. bacteriophora* [52]. Our current results indicate that *H. bacteriophora* EVs induce the TGF-β signaling Activin branch, but not the BMP branch, at low levels. Therefore, it seems that EPNs secrete distinct molecules that target both TGF-β signaling branches to interfere with the host response to nematode parasites. The precise function of the Activin branch to oppose EPN EVs will form the subject of future investigation.

The prophenoloxidase system in *D. melanogaster* participates in the defense against EPNs [56]. More precisely, the participation of PPO encoding genes was investigated in *D. melanogaster* larvae responding to *Steinernema carpocapsae* nematodes containing or lacking their associated *Xenorhabdus nematophila* bacteria. It was shown that two *PPO1* and *PPO2* genes are differentially regulated after infection with either *S. carpocapsae* symbiotic or axenic nematodes, while *PPO1*, *PPO2,* and *PPO3* genes regulate the survival of *D. melanogaster* larvae upon nematode infection. Also, a new role for *PPO3* was attributed, as *PPO3* was found to be expressed in lamellocytes and potentially in other *D. melanogaster* larval tissues upon *S. carpocapsae* infection. Here, we expand these findings by showing that *H. bacteriophora* infection factors induce *PPO* gene expression in the larval stage of *D. melanogaster*; however, the expression levels of all three *PPO* genes were particularly low in larvae treated with the *H. bacteriophora* EVs. Also, *PPO3* was barely induced by both *H. bacteriophora* infection factors (excreted–secreted products and EVs). These findings suggest that *H. bacteriophora* EVs may specifically target the PPO cascade, and *PPO3* is likely critical for *D. melanogaster’s* defense against EPN infection factors. Also, the difference in *PPO* gene expression patterns between *S. carpocapsae* and *H. bacteriophora* infections within the same host (*D. melanogaster* larvae) suggests potential differences between EPN infection strategies in relation to the PPO system. These possibilities remain to be further explored in the future.

## 5. Conclusions

Here, we present evidence that *H. bacteriophora* EVs can modulate the activity of immune-related signaling pathways in the insect model *D. melanogaster*. The broad immunomodulatory effect presumably renders adult flies sensitive to these nematode effector molecules, as shown by their decreased survival in response to the injection of *H. bacteriophora* EVs. The exact functions that are compromised in *D. melanogaster* due to reduced signaling activity remain to be explored. Therefore, future studies will deal with characterizing the impact of *H. bacteriophora* EVs on cellular immune reactions and the melanization response, which participates in the insect host anti-nematode defense [9,48,57,58,59,60]. Also, analyzing the *D. melanogaster* tissue-specific immune signaling activity against EPN EVs and the effect of the latter on the fly microbiome will provide more details on their mode of action and interaction with fundamental host physiological processes. Alternatively, fly mortality associated with HB-EV may be independent of modulation of the fly immune system and rather related to the secretion of toxins by *H. bacteriophora*. Of note, the EPNs *S. carpocapsae* and *S. feltiae* are able to produce an assortment of venom proteins with lethal properties against insects [61,62]. Such lethal proteins are yet to be discovered in *H. bacteriophora*, and future research may focus on the identification and characterization of EPN toxins and the insect tissues they target upon their release. An intriguing question to answer will be to determine all differences in immunomodulation between *H. bacteriophora* ESPs and EVs. Identifying novel effectors with insecticidal activity in EPNs would provide us with extra tools for designing alternative ways for the effective control of destructive insects. Finally, elucidating the mode of function of EPN EVs may provide clues for conserved infection mechanisms in human parasitic nematodes, which may lead to the utilization of EVs in a therapeutic context.

## Figures and Tables

**Table 1:** Primer pairs and their forward/reverse sequences utilized in quantitative RT-PCR assays.

Gene	Forward Primer ^1^	Reverse Primer ^1^
*Attacin-A*	CAATGGCAGACAATCTGG	ATTCCTGGGAAGTTGCTGTG
*Cecropin-A1*	TCTTCGTTTTCGTCGCTCTC	CTTGTTGAGCGATTCCCAGT
*Diptericin*	ACCGCAGTACCCACTCAATC	CCCAAGTGCTGTCCATATCC
*Drosomycin*	TCTTCGTTTTCGTCGCTCTC	CTTGTTGAGCGATTCCCAGT
*Metchnikowin*	TCTTGGAGCGATTTTTCTGG	AATAAATTGGACCCGGTCTTG
*Defensin*	CGCATAGAAGCGAGCCACATG	GCAGTAGCCGCCTTTGAACC
*Turandot A*	GAAGATCGTGAGGCTGACAAC	GTCCTGGGCGTTTTTGATAA
*Turandot M*	GCTGGGAAAGGTAAATGCTG	AGGCGCTGTTTTTCTGTGAC
*Basket*	GACAGCTCAGCACCAACACT	GCTTGGCATGGGTTACATTT
*Puckered*	GGCCTACAAGCTGGTGAAAG	AGTTCAGATTGGGCGAGATG
*Dawdle*	GGTGGATCAGCAGAAGGACT	GCCACTGATCCAGTGTTTGA
*Decapentaplegic*	CCTTGGAGCCTCTGTCGAT	TGCACTCTGATCTGGGATTTT
*Glass bottom boat*	CCAGATGCAGACCCTGTACAT	CTGGTGCGATGATCCAGTC
*Activin-β*	ACGGCAAATTTTGACAAAGC	TTGGTATCATTCGTCCACCA
*Prophenoloxidase 1*	CAACTGGCTTCGTTGAGTGA	CGGGCAGTTCCAATACAGTT
*Prophenoloxidase 2*	CCCGCCTATACCGAGA	CGCACGTAGCCGAAAC
*Prophenoloxidase 3*	GGCGAGCTGTTCTACT	GAGGATACGCCCTACTG
*RpL32*	GATGACCATCCGCCCAGCA	CGGACCGACAGCTGCTTGGC

^1^ Sequences are given from 5′ to 3′.

## Data Availability

The data supporting the findings of this study are available within the article.
